# Genetic Algorithm for Multiple Bus Line Coordination on Urban Arterial

**DOI:** 10.1155/2015/868521

**Published:** 2015-01-13

**Authors:** Zhen Yang, Wei Wang, Shuyan Chen, Haoyang Ding, Xiaowei Li

**Affiliations:** ^1^Jiangsu Key Laboratory of Urban ITS, Southeast University, 2 Si Pai Lou, Nanjing 210096, China; ^2^Jiangsu Province Collaborative Innovation Center of Modern Urban Traffic Technologies, 2 Si Pai Lou, Nanjing 210096, China

## Abstract

Bus travel time on road section is defined and analyzed with the effect of multiple bus lines. An analytical model is formulated to calculate the total red time a bus encounters when travelling along the arterial. Genetic algorithm is used to optimize the offset scheme of traffic signals to minimize the total red time that all bus lines encounter in two directions of the arterial. The model and algorithm are applied to the major part of *Zhongshan North* Street in the city of Nanjing. The results show that the methods in this paper can reduce total red time of all the bus lines by 31.9% on the object arterial and thus improve the traffic efficiency of the whole arterial and promote public transport priority.

## 1. Introduction

In recent years, bus priority strategy as a possible way to solve urban traffic problems has received more and more attention in China. Bus transit plays a critical role in the whole urban traffic system due to its large capacity; thus if the operation efficiency of bus transit is increased, the quality of the whole urban traffic system will rise to a new level [1, 2].

Bus priority strategy can be mainly classified into two kinds: active priority strategy and passive priority strategy. Active priority strategy can make response to the bus presence in real-time. The examples of this kind of strategy are green extension, red truncation, phase insertion, and phase rotation [3]. Passive priority strategy does not operate in real-time and is based on the regular movement of bus streams. With respect to active priority strategy, passive priority strategy has the advantages of less investment and easier operation. When the bus percentage in the urban arterial is large, passive priority strategies can always have a very significant effect [4].

In the previous studies of passive priority strategies, Khasnabis and Rudraraju (1997) found that optimization of bus headway can reduce the delay time along the bus route [5]. Skabardonis (2000) proposed a set of passive bus priority strategies by using TRANSYT-7F [6]. Zhang (2003) studied intersection phase design and timing optimal methods aiming at minimizing average delay per person at signalized intersection. As buses have larger capacity than ordinary cars, these methods can have a promotion on bus priority [7]. Ma and Yang (2007) depicted the relationship between the departure frequency of a bus line, cycle length of signalized intersection, and the number of different signal status and found that the number of different signal states can affect the average bus delay [8].

Another kind of passive bus priority strategy is bus coordination. Coordination is an effective way to improve the overall efficiency of traffic along the arterial that cannot be achieved by single traffic signal control. There are two commonly used objectives of coordination. One is to provide maximum bandwidth for vehicles along the arterial, and the other is to minimize total delays, number of stops, or queue length of the arterial system [9, 10]. As for bus coordination, it refers to making coordination schemes mainly according to bus operating parameters. However, there are not many researches in this respect. Estrada et al. (2009) provide a simulation methodology for minimizing the bus travel time by modifying the relative offset of green phase at the intersections [11].

Moreover, not much of the previous literature of passive bus priority has specially considered the factor of multiple bus lines on urban arterial. In fact, it is common to set multiple bus lines on urban arterials in China. Every bus line may have different operation characteristics. For example, a big-station bus has fewer stopping spots and thus costs shorter travel time on the arterial. This will increase the difficulty and complexity of bus passive priority control. Also, most of the previous literature about passive bus priority uses simulation methods. Analytical models have not yet received much attention.

With these reasons, this paper will focus on coordination of multiple bus lines on urban arterial. The primary objective of this paper is to reduce bus delay time and raise the operation efficiency of all bus lines on urban arterial. The main contents of this paper are (1) to define and analyze bus travel time on road section with the effect of multiple bus lines, (2) to formulate an analytical model to calculate total red time a bus encounters when travelling along the arterial according to bus travel time of each road section and timing parameters of each traffic signal, and (3) to optimize the offset scheme of traffic signals to minimize the total red time that all bus lines encounter in two directions of the arterial by using genetic algorithm.

## 2. Data Site

**Figure 1:**
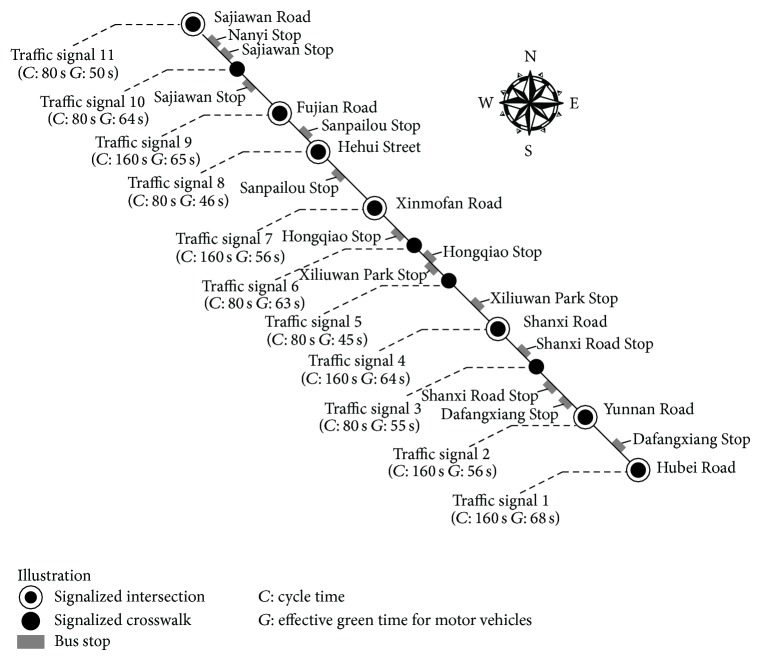
Information about the object arterial.

The data used in this paper is collected from* Zhongshan North* Street in the downtown area of Nanjing.* Zhongshan North* Street is a northwest (or southeast) direction arterial that provides a shorter route between urban area and suburban area in Nanjing; thus it is in an important geographical position. This paper selects a major part of* Zhongshan North* Street as the object arterial of this study which contains 11 traffic signals, including signalized intersections and signalized crosswalks. There are totally 7 bus lines operating through the object arterial: Line 16, Line 31, Line 34, Line 100, Line 151, Line 168, and Line d2. The structure, timing parameters of each signal, and other information of the object arterial are shown in Figure 1.

## 3. Bus Travel Time on Road Section

### 3.1. Definition of Bus Travel Time on Road Section

**Figure 2:**
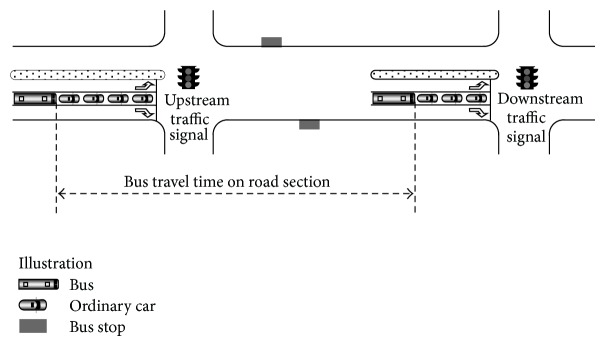
Illustration of bus travel time on road section.

To be convenient, “section” is used to call the road space between two adjacent traffic signals. Bus travel time on road section in this paper is defined as follows: the time a bus consumed from the end of vehicle queue at the upstream traffic signal to the end of vehicle queue at the adjacent downstream traffic signal (not counting the red time at the upstream signal), as illustrated in Figure 2. This definition is based on the assumption that the upstream signal is unsaturated and it describes the common situation that buses share roadway with other vehicles. It is also applicable to the situation when there is a bus exclusive lane or bus-only approach simply by replacing vehicle queue with bus queue.

It can be seen from Figure 2 and the definition that bus travel time on road section is comprised of three parts: (1) queue clearance time at upstream traffic signal, including the propagation time of backward shock wave when the signal turns green and the time a bus moves forward following other vehicles, (2) running time on the section, and (3) bus dwell time (if there are bus stops on the section).

### 3.2. Analysis of Bus Travel Time on Road Section

The data of bus travel time collected from* Zhongshan North* Street is listed in Table 1. Bus stops included by each bus line are listed in Table 2. In Table 1, “sec *i*” refers to the road space between traffic signal *i* and traffic signal *i* + 1  (*i* = 1,2,…10). The data is collected through on-board investigation between 14:30 to 16:00 on Tuesday, Wednesday, and Thursday because traffic characteristics in urban streets are similar in these three weekdays. Also during the investigation period, all the intersections and crosswalks in* Zhongshan North* Street are unsaturated. The peak hours are improper for bus coordination because some of the intersections on* Zhongshan North* Street are saturated or oversaturated, such as Shanxi Road and Xinmofan Road. Therefore peak hours are improper for bus coordination and not chosen in this study.

It can be noticed from Table 1 that bus travel time on each road section varies from one bus line to another. The reasons lie in these aspects: (1) passenger demands varying from one bus line to another will lead to different bus dwell time; (2) different vehicle conditions of each bus line will lead to different running time; (3) different number of bus stops included by each bus line will lead to different total bus dwell time (as shown in Table 2). For example, Line d2 is a big-station bus line and only stops at* Sanpailou* and* Xiliuwan Park* on the object arterial, while Line 31 has nearly all the bus stops listed in Table 2.

Another fact that can be noticed from Table 1 is that travel time of a bus line on each section varies from one direction to another. That's because buses share roadway with other vehicles on the object arterial; thus the imbalance of traffic volume in two directions will affect bus travel time. Also, the asymmetric layout of bus stops will also cause directional difference of bus travel time on the section. For example, as shown in Figure 1,* Shanxi Road* Stop in the direction from southeast to northwest is set on sec 3 while the opposite stop is set on sec 2.

## 4. Bus Time Calculation Model at Traffic Signal

In this paper, bus time at traffic signal refers to the red time a bus encounters when arriving at the end of vehicle queue at this signal. During the red interval, a bus has to wait until the green signal starts and thus delays will occur. The amount of red time that a bus consumes at a signal is related to the signal timing parameters (including cycle length, green phase time, and the offsets along the arterial) and total travel time prior to this signal.

As for the coordination, cycle length of each traffic signal on the arterial needs to be the same or have multiple relationships at least to ensure the unique offset. In this paper, offset of signal *i* refers to the difference of green start time between signal *i* and the first signal (*i* = 1,2,…11). There are mainly two kinds of coordination: delay (or number of stops, maximal queue length) minimization and bandwidth maximization as mentioned in the previous paragraphs. In this paper, it is hard to provide bandwidth for every bus line due to the variety of bus travel time (as shown in Table 1). Therefore, delay minimization method is used. A model is formulated in the following paragraphs to calculate the total red time a bus encounters when travelling along the arterial according to bus travel time on each road section and timing plan of each traffic signal.

**Figure 3:**
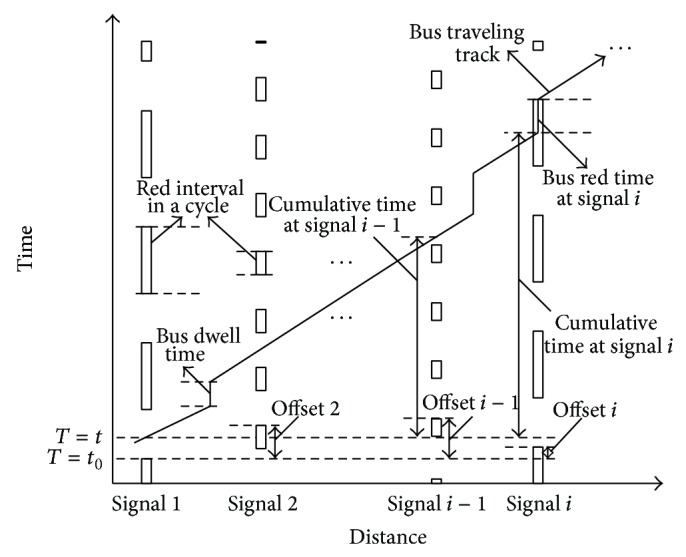
Illustration of bus time calculation model at traffic signal.

Figure 3 illustrates the travelling track of a bus in one direction. Timing plan and offset of each traffic signal are also displayed for red time calculation. Cumulative time CT_*i*_ is used to denote the total travel time prior to signal *i*, including total bus travel time on previous road section and total red time at previous signals. Thus cumulative time CT_*i*_ can be calculated as follows (CT_1_ = 0):

(1) $$\begin{array}{c} \text{C}{\text{T}}_{i}=\overset{i-1}{\underset{k=1}{\sum }}\left(T_{k}+r_{k}\right), \end{array}$$

where *T*_*k*_ refers to the bus travel time on previous section *k* and *r*_*k*_ refers to the red time a bus encounters at previous signal *k*.

To calculate *r*_*i*_, a nonnegative integer *n* needs to be found to satisfy

(2) $$\begin{array}{c} O_{i}+\left(n-1\right)*C_{i}<\text{C}{\text{T}}_{i}+t-t_{0}\leq O_{i}+n*C_{i}, \end{array}$$

where *C*_*i*_ is the cycle length of signal *i*, *O*_*i*_ is the offset of signal *i*  (0 ≤ *O*_*i*_ < *C*_*i*_), *t*_0_ is the green starting time of signal 1 in the travelling direction of the bus, and *t* is the bus arrival time at signal 1  (0 ≤ *t* − *t*_0_ < *C*_1_). Then *r*_*i*_ can be calculated as

(3) $$\begin{array}{c} r_{i}=\left\{\begin{array}{c} O_{i}+n*C_{i}-\text{C}{\text{T}}_{i}+t-t_{0}\\ 0\leq O_{i}+n*C_{i}-\text{C}{\text{T}}_{i}+t-t_{0}\leq C_{i}-G_{i}\\ 0\\ C_{i}-G_{i}<O_{i}+n*C_{i}-\text{C}{\text{T}}_{i}+t-t_{0}<C_{i}, \end{array}\right. \end{array}$$

where *G*_*i*_ is the effective green time of signal *i*.

It can be seen that the calculation of red time a bus encounters at each signal is such an process: red time *r*_1_ is first used to calculate the cumulative time CT_2_; then the red time *r*_2_ at signal 2 can be calculated according to CT_2_, and so forth. The total red time *R* of a bus along the arterial can be expressed as

(4) $$\begin{array}{c} R=\underset{i}{\sum }r_{i}. \end{array}$$

The total red time *R* of a bus will change with bus arrival time *t* at the first signal; therefore *R* can be expressed as a function of *t*; that is, *R* = *R*(*t*). Suppose the bus arrives with equal probability at any time at the first signal; then the average total red time $\overset{-}{R}$ of a bus along the arterial can be calculated as

(5) $$\begin{array}{c} \overset{-}{R}=\frac{\left(\sum_{i=0}^{C_{1}}R\left(t_{i}\right)\right)}{C_{1}}. \end{array}$$

When adding the factors of bus driving direction and multiple bus lines, the total red time calculation model can be expressed as

(6) $$\begin{array}{c} R_{T}=\overset{N_{b}}{\underset{i=1}{\sum }}\left({\overset{-}{R}}_{i,\text{outbound}}+{\overset{-}{R}}_{i,\text{inbound}}\right), \end{array}$$

where *R*_*T*_ is the total red time of all bus lines in two directions (outbound and inbound) of the arterial, *N*_*b*_ is the total number of bus lines. It can be seen from (1)~(6) that when the bus travel time on each road section and the timing plan of each traffic signal are determined, *R*_*T*_ is only the function of offset scheme of signals along the arterial.

## 5. Genetic Algorithm

### 5.1. Advantages of Genetic Algorithm

Genetic algorithm (GA) as an intelligent optimization method has already been widely used in the area of traffic control. In this paper, GA is used to optimize the offset scheme of traffic signals along the arterial to achieve minimum total red time *R*_*T*_ for all bus lines. Compared to traditional optimization algorithms, GA has the following advantages:

1. GA does not require the solving model to be explicitly expressed. It only needs the fitness function and related variables. For the problem this paper studies, it is hard to get the explicit expression of total red time *R*_*T*_. Thus the optimal value of *R*_T_^*^ cannot be easily obtained by traditional optimization methods. However, the only factor that will affect this total red time is the offset scheme of signals along the arterial. Therefore the problem of this paper can be solved by GA.
2. The complexity of GA is approximately linearly correlated with the scale of the problem so that it will not cause the dimension disaster. The problem of this paper contains 11 traffic signals and the combination quantity of the offsets is large. Therefore GA is very suitable for solving this problem.

In the previous research of GA in the area of traffic control, Chang and Peng (2003) analyzed the real departure-arrival model of traffic flow at intersection and developed a new method for urban arterial coordinate control based on genetic algorithm [12]. Singh et al. (2009) developed a traffic signal control strategy by using genetic algorithms which can provide optimum green time extensions and optimize signal timings in real time [13]. Yu et al. (2010) put forward a bilevel programming model in which passenger assignment model is the lower level and bus frequency optimization model is the upper level and then use genetic algorithm mainly to obtain the optimal bus frequencies to minimize the total travel time of passengers [14].

### 5.2. Algorithm Design

Generally speaking, the design of GA can be divided into five steps: chromosome coding, determination of fitness function, determination of select strategy, determination of genetic operator, and selection of the control parameters. First the initial population is generated through the chromosome coding of decision variables. Then proper select strategy is used to choose elite individuals in the current population according to their fitness. These elite ones will be the parents of the next generation. After that, genetic operators are applied to the parents to create the next generation. This process will continue until the terminal conditions are met.

(*1) Chromosome Coding*. Eight-bit binary coded string is used to denote the offset of each traffic signal shown in Figure 1 (except the first signal). This encoding method fits the problem of this paper because 8-bit binary coded string can denote every integer between 0 and 256, and the biggest cycle length is only 160 s. The binary string of each signal is combined into a chromosome, and then the initial population is generated.

A chromosome is also called genotype individual. Each genotype individual of every generation can be expressed as

(7) xlk=b2,1,b2,2,…,b2,8,…,…,bi,j,…,…,  bn,1,bn,2,…,bn,8b2,1,b2,2,…,b2,8,…,…,bi,j,…,…,,

where *x*_*l*_^*k*^ is genotype individual *l*  (*l* = 1,2,…*N*, and *N* refers to the population size) in generation *k*  (*k* = 1,2,…*N*_0_, and *N*_0_ refers to the maximum number of generations), *b*_*ij*_ is binary number of signal *i* in position *j*, and *n* is the total number of signals. To decode this genotype individual to phenotype, that is, offset, the following equations are used (when *i* = 2,…11):

(8) $$\begin{array}{c} O_{i}=\left\{\begin{array}{cc} \mathrm{int}\left(\frac{160}{2^{8}}\cdot \overset{8}{\underset{j=1}{\sum }}b_{i,j}\cdot 2^{8-j}\right), & C_{i}=160\\ \mathrm{int}\left(\frac{80}{2^{7}}\cdot \overset{8}{\underset{j=2}{\sum }}b_{i,j}\cdot 2^{8-j}\right), & C_{i}=80. \end{array}\right. \end{array}$$

When *i* = 1, *O*_1_ = 0.

(*2) Determination of Fitness Function*. The minimization of total red time *R*_*T*_ mentioned in the previous texture is chosen as the objective function. The fitness of each individual in the population is directly taken as the value of objective function; that is, *R*_*T*_ = *f*(*O*_1_, *O*_2_,…*O*_*n*_).

(*3) Determination of Select Strategy*. Breeding pool selection is chosen as the select strategy. First, elite individuals whose fitness ranks the top 20% of every generation are selected to form a breeding pool (these elite individuals directly go into the next generation). Elite ones in the breeding pool are randomly extracted in pairs to generate new individuals through crossover or mutation until the population of the next generation reaches the upper limit.

(*4) Determination of Genetic Operator*. Crossover operator and mutation operator are chosen to generate new individuals. Crossover operator is a function that breaks up two of the genotype individuals into four and combines them in some new way. Mutation operator is a function that makes small and random changes to an existing genotype individual [15]. 

(*5) Selection of the Control Parameters*. The main parameters of the algorithm are chosen as follows: *N* = 100, *N*_0_ = 100, the probability of crossover *p*_*c*_ = 0.8, the probability of mutation *p*_*m*_ = 0.2, and elite proportion of every generation *p*_*e*_ = 0.2. When the number of generations reaches *N*_0_, the algorithm terminates.

### 5.3. Result of Genetic Algorithm

**Figure 4:**
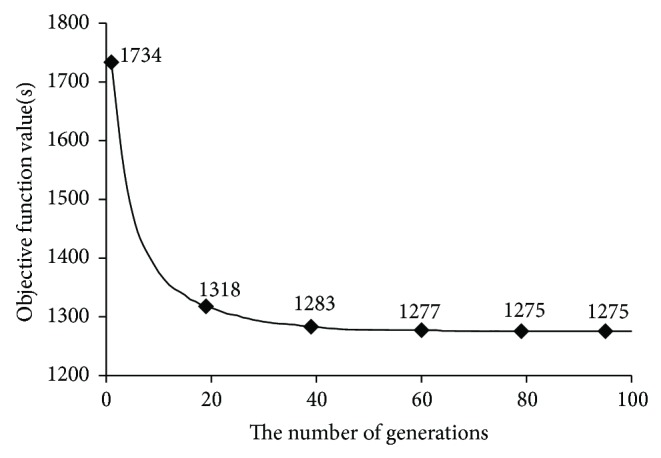
Average trend of objective function value.

To solve the problem of coordination control for multiple bus lines in this paper, a procedure of GA has been developed with python 3.0 and runs for 60 times. The average trend of objective function value (total red time *R*_*T*_) is shown in Figure 4 and the distribution of optimal objective function values is listed in Table 3.

Figure 4 illustrates that the algorithm has a fast convergence rate. The objective function value has already been close to the optimal value after the 20th generation of the population. Table 3 shows that it is difficult for the algorithm to find the exact globally optimal solution. The reason may lie in the large scale of problem in this paper. However, it does not prevent the algorithm from finding a satisfactory solution. The minimum of optimal objective function values among the outputs of the procedure is 1180, and the corresponding offset scheme of traffic signals on the object arterial is [0,82,29,0, 63,40,136,43,142,15,41] (from signal 1 to signal 11 shown in Figure 1). Compared to the initial stage of optimization, when the offset of each signal along the arterial is randomly chosen, the average objective function value (i.e., total red time) is as high as 1734. If the current offsets on* Zhongshan North* Street are randomly set, the total red time of all bus lines can be reduced by 31.9% as shown in Table 3.

## 6. Conclusions

This paper analyzed the characteristic of bus travel time on road section with the effect of multiple bus lines and formulated a bus time model at traffic signals to calculate the total red time a bus encounters when travelling along the arterial and used genetic algorithm to optimize the offset scheme of traffic signals on the major part of* Zhongshan North* Street in the city of Nanjing. The optimization objective is to minimize the total red time of all the bus lines in two directions of the street. The results show that the model and algorithm can reduce the total red time by 31.9% on the arterial.

The methods in this paper only need to collect bus travel time and make a proper design of the offsets in the arterial, without having to change other signal timing parameters of intersections and crosswalks and thus can be easily operated and extensively applied. The model and algorithm can also be extended to solve the problem of bus coordination in the network if a few alterations are made. However, the stochastic characteristic of bus travel time on road section for each bus line is not considered in this paper due to lack of data. This work will be conducted in the future study.

## Figures and Tables

**Table 1:** Bus travel time of all the bus lines on each section.

Bus line	Bus travel time(s) (from southeast to northwest)									
	Sec 1	Sec 2	Sec 3	Sec 4	Sec 5	Sec 6	Sec 7	Sec 8	Sec 9	Sec 10
Line 16	69	39	63	73	60	20	99	74	37	57
Line 31	62	36	53	90	55	20	110	60	38	80
Line 34	105	46	69	95	66	28	97	77	33	49
Line 100	76	51	65	40	51	23	60	69	31	69
Line 151	72	49	72	62	57	17	66	86	43	64
Line 168	61	38	60	19	66	28	84	73	37	84
Line d2	48	41	42	49	46	19	72	60	32	41

Bus line	Bus travel time(s) (from northwest to southeast)									
	Sec 1	Sec 2	Sec 3	Sec 4	Sec 5	Sec 6	Sec 7	Sec 8	Sec 9	Sec 10

Line 16	58	63	35	29	110	64	89	51	45	39
Line 31	77	107	67	31	207	118	116	41	65	40
Line 34	108	57	34	26	67	56	78	26	51	33
Line 100	92	151	22	24	43	86	103	42	63	64
Line 151	52	69	51	34	99	62	63	38	34	36
Line 168	39	85	60	30	95	62	107	59	62	31
Line d2	44	55	66	22	57	21	69	45	22	33

**Table 2:** Bus stops included by each bus line.

Bus line	Name of bus stops (from southeast to northwest)						
	*Dangfangxiang *	*Shanxi Road *	*Xiliuwan Park *	*Hongqiao *	*Sanpailou *	*Sajiawan *	*Nanyi *
Line 16	✓	—	✓	✓	✓	✓	—
Line 31	✓	✓	✓	✓	✓	✓	✓
Line 34	✓	✓	✓	✓	✓	✓	—
Line 100	✓	✓	—	✓	✓	✓	✓
Line 151	✓	✓	✓	✓	✓	✓	—
Line 168	✓	—	✓	✓	✓	✓	✓
Line d2	—	—	✓	—	✓	—	—

Bus line	Name of bus stops (from northwest to southeast)						
	*Dangfangxiang *	*Shanxi Road *	*Xiliuwan Park *	*Hongqiao *	*Sanpailou *	*Sajiawan *	*Nanyi *

Line 16	✓	—	✓	✓	✓	✓	—
Line 31	✓	—	✓	✓	✓	✓	—
Line 34	✓	✓	✓	✓	✓	✓	—
Line 100	✓	✓	—	✓	✓	✓	—
Line 151	✓	—	✓	✓	✓	✓	—
Line 168	✓	—	✓	✓	✓	✓	—
Line d2	—	—	✓	—	✓	—	—

**Table 3:** Distribution of optimal objective function values and the benefits.

Range	1180~1200	1201~1250	1251~1300	1301~1350	1351~1400	1401~1500
Occurrence number	8	21	14	8	4	5

Minimum optimal value of objective function among the outputs				1180		

Average objective function value under random offsets				1734		

Reduced rate				31.9%		
